# Nutritive quality prediction of peaches during storage

**DOI:** 10.1002/fsn3.2287

**Published:** 2021-05-27

**Authors:** Yuming Zhong, Yao Bao, Yumin Chen, Dequan Zhai, Jianliang Liu, Huifan Liu

**Affiliations:** ^1^ College of Environmental Science and Engineering Zhongkai University of Agriculture and Engineering Guangzhou China; ^2^ College of Light Industry and Food Zhongkai University of Agriculture and Engineering Guangzhou China; ^3^ Modern Agriculture Research Center Zhongkai University of Agriculture and Engineering Guangzhou China; ^4^ Guangzhou Key Laboratory for Research and Development of Crop Germplasm Resources Zhongkai University of Agriculture and Engineering Guangzhou China; ^5^ Guangdong Provincial KeyLaboratory of Lingnan SpecialtyFood Science and Technology Guangzhou China

**Keywords:** Fourier transform‐near infrared spectroscopy, nutritive quality, peach, random forest, shelf‐life

## Abstract

Peaches (*Prunus persica* L. Batsch) are commonly consumed fruits with high nutritional value. We evaluated the nutritive qualities of peach fruit during storage. Heatmap analysis showed that protein, ash, and crude fiber contents clustered together, whereas fat and reducing sugars clustered separately. We then classified the nutrients into two clusters; cluster 1 showed low fat and reducing sugar levels and high protein, crude fiber, and ash levels, whereas cluster 2 showed high fat and reducing sugar levels and low protein, cruder fiber, and ash levels. Partial least squares regression and random forest analyses showed accuracies of 67% and 61%, respectively. Spectra at 1,439 and 1,440 nm indicated reducing sugars, and the spectrum at 2,172 nm indicated protein. Thus, Fourier transform‐near infrared spectroscopy could predict the two clusters based on five nutritive qualities. Our findings may help to establish guidelines for promoting the acceptability of peach fruits among consumers.

## Graphical abstract

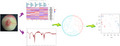

## 1 INTRODUCTION

Peach (*Prunus persica* L. Batsch) is the third most important deciduous tree fruit worldwide owing to its tender texture, pleasant flavor, and nutrient content (Zhang et al., 2010). The nutrients found in peach fruit include dietary fiber, minerals, proteins, and soluble sugars, among others, providing the fruit with health benefits against chronic diseases, including cardiovascular disease and certain types of cancer (Gil et al., 2002). Furthermore, the nutritional properties of peaches, as one important indicator of peach fruit quality, provide other health effects (Rodríguez‐González et al., 2018; Zhang et al., 2010). Although the nutritional quality of fruit is a complex feature, the attributes that confer health benefits to fruits have not yet been fully elucidated (Alvarezsuarez et al., 2014). In contrast to specific micronutrients (e.g., minerals and vitamins), the contents of nonessential compounds, such as dietary fibers, have not been clear indicators of nutritional quality. Commercially, the definition of quality has focused more on consumer demand to increase peach consumption (Crisosto et al., 2008).

Many fruit quality attributes affecting consumer acceptance and price are still tested using traditional approaches. The most commonly used method to assess macronutrient content is a chemical method, which is time‐consuming, expensive, and destructive (Ruiz et al., 2008). With the development of various technologies, near infrared (NIR) spectroscopy has been shown to have the advantages of rapid and nondestructive analysis (Pissard et al., 2013). NIR radiation covers the range from 380 to 2,500 nm in the electromagnetic spectrum, and the signals of most major structures and functional groups of organic compounds can be detected using stable spectrograms. When obtaining spectra, chemometric methods, which involve spectral pretreatments and regression methods, are applied to extract information related to quality attributes and to eliminate the interference of factors irrelevant to sample concentration. Therefore, NIR has been used to determine internal quality attributes of various fruits, such as kiwifruits and mangoes (Jin et al., 2012; Subedi et al., 2007).

Because NIR can be used for qualitative and quantitative analysis of vital information, nondestructive methods are preferred for analysis of peach fruits. Liu et al. established a rapid diffuse reflectance NIR spectroscopy (DR‐NIR) method along with chemometric techniques for clear classification of two varieties of peach kernels. The correlation coefficients of two calibration models were above 0.99, and the root‐mean‐square error of deviation of linoleic and oleic acids were 1.266% and 1.412%, respectively, indicating that DR‐NIR combined with principal component analysis and partial least squares regression (PLS) could be used efficiently to identify and quantify peach kernels (Liu et al. (2014). Takano et al. (2007) evaluated polyphenols in peach using NIR spectroscopy in the 1,100–2,500 nm wavelength range, and a rough prediction was achieved, with an *r* value of 0.80, in which spectral peaks at 1,720 nm were shown to correlate with polyphenol content by multiple regression analysis. Liu et al. (2014) and Shao et al. (2011) used Fourier transform (FT)‐NIR reflectance spectroscopy in the 928–2,331 nm waveband to measure the valid acidity (pH) of peaches using a PLS model and least squares support vector machine (LS‐SVM) model built. A supervised machine learning model was used to predict the quality. For example, successful models, including based LS‐SVM with a radial basis function kernel, were built based on the application of NIR spectroscopy for online quantitative monitoring of soluble solid content and concentrations of danshensu, protocatechuic aldehyde, hydroxysafflor yellow A, and salvianolic acid B (Jin et al., 2013). The results indicated this spectroscopy method was suitable for the prediction of the acidity of peaches (Shao et al., 2011).

Therefore, in this study, we aimed to employ FT‐NIR spectroscopy to predict the nutritive quality of peaches. Our findings may promote the acceptability of peaches to consumers and increase peach consumption.

## 2 MATERIALS AND METHODS

### 2.1 Materials

On the day of harvest, peach fruits of uniform commercial maturity (fruit hanging time of approximately 75 days) with no disease or mechanical wounding were selected by experienced farmers and transported to the laboratory. In total, 87 peach fruits were obtained. Sulfuric acid was purchased from Wuxi Jiahong Chemical Trading Co. Ltd. Ultrapure water was produced using a Milli‐Q system. Folin‐phenol reagent was purchased from Sangon Biotech Co., Ltd. All other chemicals and reagents used were of the highest analytical grade commercially available.

### 2.2 FT‐NIR spectra acquisition

The spectra were collected using an Antaris II FT‐NIR analyzer (Thermo Fisher Scientific). The spectrometer was equipped with a DSP electromagnetic interferometer, optical table, InGaAs detector electronic control technology, and sampling module (including a transmission analyzer module, tablet analyzer module, ISAM, and FOAM). Diffuse reflectance spectra were obtained over the range of 4,000–0 cm^−1^ (1,000–2,500 nm) at a spectral resolution of 4 cm^−1^ with 50 scans/spectrum. Spectral acquisition involved ISAM for scanning from peach fruit pulp after peeling and beating, compiled into one spectrum; this spectrum was set as the characteristic spectrum of the sample. All NIR data were obtained using first derivative and Norris first derivative pretreatment methods (Pissard et al., 2013; Wang et al., 2015).

### 2.3 Determination of nutrition content

The nutrient contents, including reducing sugars, were evaluated according to the phenol‐sulfuric acid method. The protein, fat, crude fiber, and ash contents were evaluated by the GB 5,009.5‐2016 (Shuo et al., 2019), GB 5,009.6‐2016 (called the second method) (Piao‐Ping et al., 2019), GB/T 5,009.10‐2003 (Wang et al., 2013), and GB 5,009.4‐2016 (called the first method) (Liang‐Jun et al., 2018) methods, respectively.

### 2.4 Nonparametric multivariate statistical tests and matrix plots

Nonparametric multivariate analysis of variance (Adonis) and the multi‐response permutation procedure (MRPP) were used for the analysis of the overall peach datasets. The datasets were preprocessed by the “Euclidean” distance method (Anderson, 2001; Clarke, 2010; Legendre, 2012; Warton et al., 2012). Kruskal–Wallis tests were used to analyze the differences between two clusters.

Heatmap, density plot, and matrix plot analyses were used to evaluate different nutritive quality levels of peaches. Heat maps were calculated from the “Euclidean” distance using the R package complexheatmap. Density plots and matrix plots were created using the R package latticeExtra and Gally.

### 2.5 K‐means clustering unsupervised model

The k‐means clustering model was used to classify nutrient levels of peaches in part II of the study. The k‐means clustering model was applied using hierarchical k‐means clustering, which combines hierarchical clustering and the k‐means methods in R package. The procedure involved computing the center (i.e., the mean) of each cluster and then performing k‐means analysis using the set of cluster centers (defined in step 3) as the initial cluster centers. The clustering was then optimized.

Peach fruit nutrients data were preprocessed using standard methods. Then, hierarchical clustering was performed, and the tree was cut using k‐clusters. Next, k‐means used k‐cluster results as centroids for each cluster. Subsequently, the distances between the peach data vector and each centroid were calculated. The k‐means was used to generate peach nutrient level classifiers, and the results were aggregated based on minimizing the within‐cluster sum of squares.

Ci is the collection of centroids in set C, and each data point *x* was assigned to a cluster based on Equation 1:

(1) $$\text{arg}\underset{\text{ci}\in c}{\text{min}}\text{dist}{\left(\text{Ci},x\right)}^{2}$$

Where dist (·) is the standard (L2) Euclidean distance. The set of data point assignments for each *i* th cluster centroid was set to Si. Then, the results of the clusters were applied for visual classification and hierarchical k‐means clustering.

The cluster number was chosen according to the average silhouette width and the fruit quality level studies (Hartigan & Wong, 1979; Kaur & Dhillon, 2015; Wang et al., 2015). All analyses were conducted by R 3.26, with factoextra, cluster packages.

### 2.6 PLS and random forest (RF) supervised machine learning model

PLS and RF models were chosen to study the relationships between NIR spectroscopy and nutrient levels in peaches. The PLS model was used to construct prediction models for the major nutrients in fruits, as reported (Sun et al., 2009). The classification model was also built to predict and select useful wavelengths under 10‐fold cross‐validation (Chong & Jun, 2005; Nicola et al., 2007; Wang et al., 2015).

Random forest was the second model chosen to predict the nutrient levels of peaches. RF is a nonparametric, nonlinear classification algorithm (Ho, 1995) that does not require data preprocessing.

The RF model enabled application of the “bootstrap strategies” proposed by Breiman (2001) by combining with the “random subspace” method. According to the analysis, the RF ensemble grows multiple unpruned trees (mtree) in bootstrap samples of peach data. Two‐thirds of the original data, known as “in‐bag” data for training, and one‐third of the original data, known as “out‐of‐bag” data for testing, were analyzed with the bootstrap method and used to build the tree. The prediction results were evaluated using trees split into many nodes according to random subsets of parameters from the peach dataset (mtry); the default mtry value was the square root of the total number of variables and was determined by the 10‐fold cross‐validation test. Index tests were also conducted using RF models. Measurement of PLS and RF model classification accuracy and kappa values were used to select the optimal model parameters and model quality. Model and cross‐validation were conducted using the R package caret.

### 2.7 Analysis processes

Preprocessed peach data were further analyzed with the following analysis processes. (a) Matrix and heatmap plots were described and used to confirm the relationships among all peach datasets and NIR datasets. (b) k‐means clusters combined with two of three nonparametric multivariate statistical tests (i.e., Adonis and MRPP) were used for the analysis of the clusters of peach fruit nutrient levels. Two clusters of nutrient levels were then obtained and verified. (c) PLS and RF models were used for tracking and studying the responses of NIR data to different nutrient levels/clusters. The model was then used to predict the final results. (d) Supervised machine learning models based on nutrient parameter matrices (such as reducing sugar, protein, and ash) were used to the link the functional structures of nutrient variables and NIR data. Models, spectrum explanations, and nutrient parameter distributions were used to elucidate the relationships between NIR and peach nutrient levels.

## 3 RESULTS AND DISCUSSION

### 3.1 Peach fruit data

**FIGURE 1:**
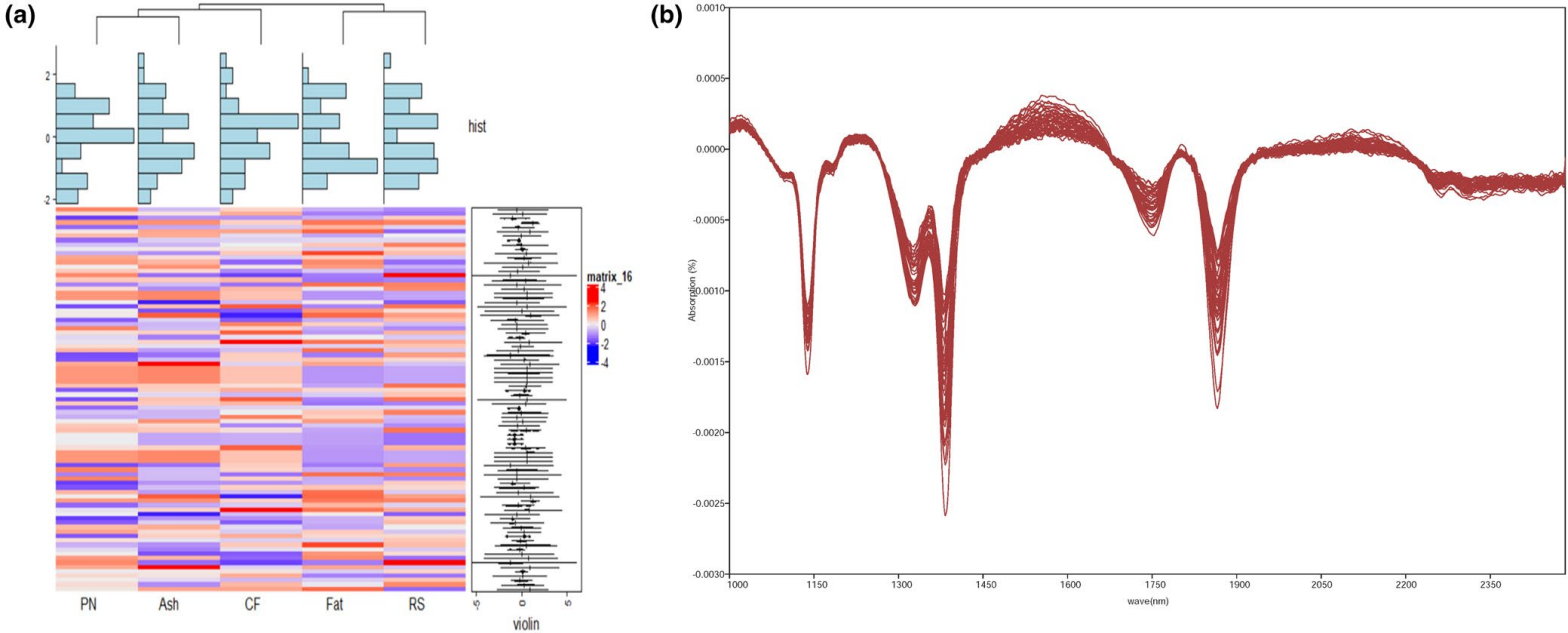
(a) Heatmap based on the peach fruits’ nutrient data, including protein (Pn), fat, ash, crude fiber (CF), and reducing sugar (RS). (b) NIR spectra of the peach fruits

In this study, the nutrient contents of 87 peach fruits were assessed; the data are shown in Table 1. In terms of the nutritive quality of peach fruit, a comprehensive heatmap analysis (Figure 1a) showed the indirect and nonvisual relationships of five types of nutrients. The protein, ash, and crude fiber contents of all 87 peach fruits clustered together, whereas the fat and reducing sugar contents formed a separate cluster, indicating possible correlations among nutrients.

**TABLE 1:** The nutritive overall data of peach fruit

Modules	Nutrients	*n*	Mean	*SD*
Nutrient	Reducing sugar (RS) (mg/L)	87	0.148	0.027
	Crude fiber (CF) (mg/L)	87	4.172	0.288
	Protein (Pn) (mg/L)	87	0.839	0.047
	Fat (mg/L)	87	0.120	0.026
	Ash (mg/g)	87	0.457	0.052

Traditional, energy‐dilute diets have been swiftly replaced by high fat, energy‐dense diets; diets with a higher energy density should include increased intake of complex proteins, minerals, and dietary fiber (Du et al., 2002; Popkin, 2009). Diets with low sugar and fat contents, high protein contents, and abundant minerals and dietary fiber are beneficial to the human body, particularly with respect to protection against chronic diseases, including cardiovascular disease and certain types of cancer (WHO, 2003). Our results of the nutrient contents of peaches, as shown in Figure 1a, could help to improve consumer acceptability of peaches, particularly for consumers who care about energy intake.

### 3.2 FT‐NIR spectroscopy data

As shown in Figure 1b, we then performed FT‐NIR spectroscopy of peach fruits and showed similar spectral tendencies, with only minor differences in a few peaks. Spectra in the FT‐NIR range contain abundant information concerning O‐H, C‐H, and N‐H vibration absorptions, making the measurement of various quality attributes of fruits possible (Pissard, 2013). In our study, there was a strong signal at 1,150 nm, indicating the second overtone C‐H stretching. The absorption peak at 1,800 nm represented the first overtone C‐H stretching. The peaks ranged from 1,300 to 1,345 nm, indicating the presence of the combination C‐H stretching (Table 2). The absorption peak at 1,745 nm represented the first overtone O‐H stretching and the first overtone N‐H stretching (Barbara, 2005). The above wavebands included typical absorption bands for some chemical groups; for example, C‐H and O‐H could represent the pH value, C‐O could represent COOH, O‐H could represent carboxyl acids, and C = O could represent saturated and unsaturated carboxyl acids to indicate acidity (González‐Caballero et al., 2010).

**TABLE 2:** Near infrared peak and characteristic structure of peach

Wavelength (nm)	Assignment
1,150	The second overtone C‐H stretching
1,300–1,345	The presence of the combination C‐H stretching
1,745	Represented the first overtone O‐H stretching and the first Overtone N‐H stretching
1,800	The first overtone C‐H stretching

Pigment content has been related to some quality attributes, including soluble solid contents and firmness. Additionally, Zude et al. (2006) found strong correlations of the peak absorbance of chlorophyll at 680 nm with harvest date (*r* = .59), background color (*r* = .74), and the starch index SI (*r* = .64). Although the specific peaks cannot represent macronutrients, Pissard et al. (2013) predicted the total polyphenol content in apples using spectra recorded in the 400–2,500 nm region.

Regarding sugars, previous studies have reported inconsistent results. For example, one study showed that O‐H represented sugars in vegetables and fruits (Rodriguez‐Saona et al., 2001). Additionally, Bureau et al. (2012) showed that different sample preparation conditions did not affect sugar concentrations. Moreover, mid‐infrared spectra showed excellent ability to predict sugar contents. Therefore, we concluded that the measurement of the peach fruit attributes, using FT‐NIR spectra, particularly for reducing sugars, required additional analyses.

### 3.3 Clusters of peach fruit nutrients

**FIGURE 2:**
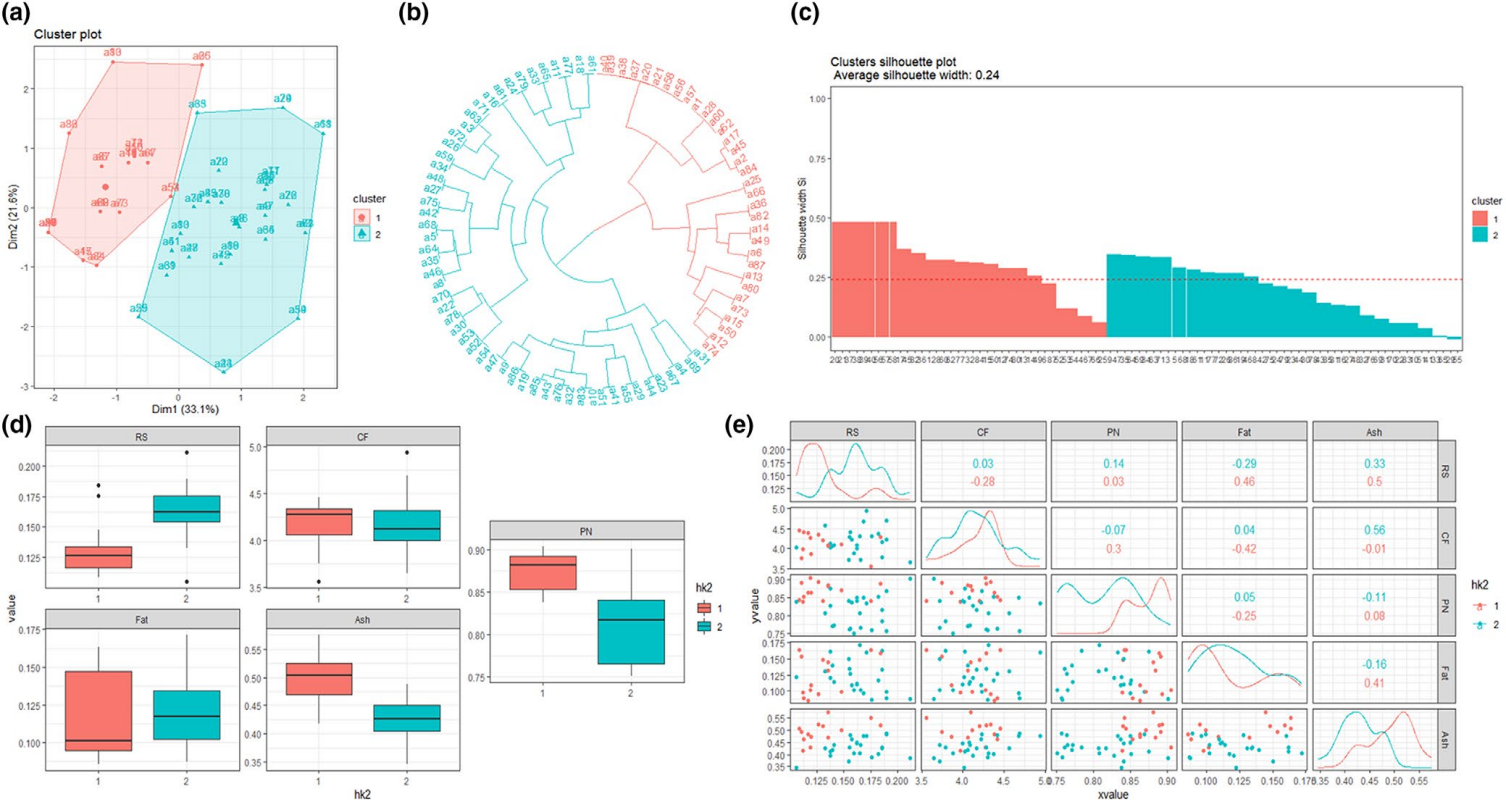
Schematic images of (a) cluster analysis, (b) taxonomy tree, (c) their average silhouette widths, (d) the box plot, and (e) matrix based on the result of k‐mean analysis of the peach fruit nutrients

For measurement of the attributes of peach fruits by FT‐NIR spectroscopy, the PLS method was first used to determine whether the spectra were correlated with the different nutrients. Our results showed that the *R* ^2^ values were .06 for reducing sugar and ash, .05 for crude fiber, .07 for protein, and .04 for fat. Therefore, the correlations of FT‐NIR spectra with the five nutrients were not obvious, potentially because of the many compounds present in the pulp of peach fruits. Subsequently, for better labeling of nutritive qualities, we classified the five nutrients in two clusters based on correlation analysis of overall data by k‐meant analysis. As shown in Figure 2a, cluster plot analysis showed that the explanation degree was 54.7%; those of the *x*‐ and *y*‐axes were 33.1% and 21.6%, respectively. Moreover, as shown in Figure 2b, the numbers of cultivars in clusters 1 and 2 were 38 and 49, respectively. The average silhouette width (Figure 2c) was 0.24, indicating the basic separation of the two clusters when clustering five nutrients together for the peach fruits. The box plot in Figure 2d shows the two clusters for each nutrient. By Adonis analysis, there were significant differences among reducing sugars, protein, and ash; the *F* values were 34.6, 38.51, and 33.6, respectively, and the *p* values were all below .01. In terms of fat and crude fiber, although the *p* values were .12 and .52, the medians showed substantial differences. Indeed, the fat contents of clusters 1 and 2 were 0.1 and 0.11 mg/ml, respectively, and the crude fiber contents were 4.26 and 4.12 mg/ml, respectively. Consistent with the results in Figure 2d, matrix analysis showed the basic separation of the five nutrients (Figure 2e).

As shown in Figure 2, cluster 1 showed low levels of fats and reducing sugars and high levels of protein, crude fiber, and ash. In contrast, cluster 2 showed high levels of fats and reducing sugars and low levels of protein, cruder fiber, and ash. When considering the quality of fruits associated with protective activities against human diseases, diets can be classified as energy‐dense or energy‐dilute (Du et al., 2002; Popkin, 2009). Because 1 g of protein, carbohydrates, and fat generates 4, 4, and 9 kcal of energy and because dietary fiber and ash are beneficial for human health, we characterized cluster 1 as low‐energy foods with a relatively rich nutritional profile and cluster 2 as energy‐dense foods with a relatively poor nutritional profile. The explanations for the two clusters of peach fruits may provide guidelines for growers and distributors to improve commercial consumption because the price of peaches depends on the quality of the cultivars.

### 3.4 Establishment of a supervised machine learning model

**FIGURE 3:**
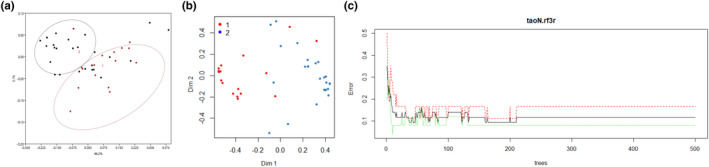
(a) The cluster analysis of the NIR spectra of the peach fruits. (b) The Random Forest prediction model based on the result of k‐mean, (c) and its error analysis

In this study, cluster analysis of the NIR spectra of peach fruits indicated two clusters (Figure 3), consistent with the clusters of nutrients in Figure 2a. In general, two supervised machine learning models, PLS and RF (Figure 3b), were established based on five nutrients and NIR spectra. The accuracies of these approaches were 67% and 61%, respectively, and the kappa values were 0.2 and 0.32, respectively. Furthermore, after 200 trees (Figure 3c), the error was stable based on the RF analysis. Thus, we concluded that from the two models, the FT‐NIR spectra of the peach fruits could predict the two clusters based on the five nutritive qualities; the relatively low accuracy may relate to the limited number of cultivars used.

**FIGURE 4:**
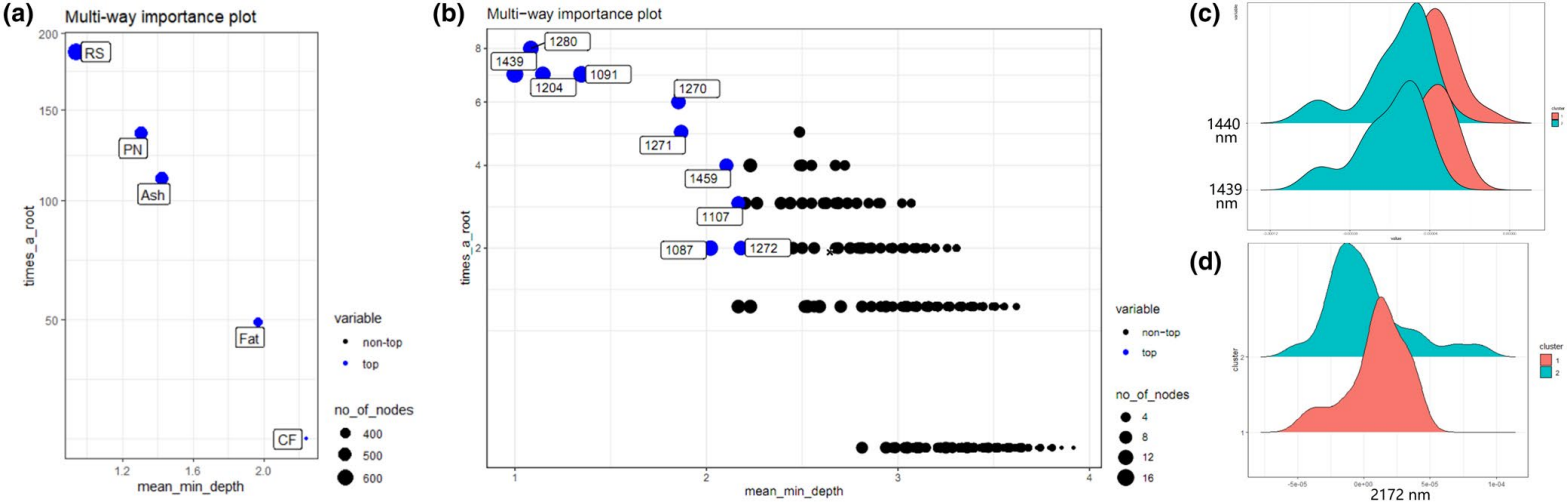
Based on the Random Forest prediction model, the images of multiway importance plot of (a) nutrients, including reducing sugar, protein, ash, fat, and crude fiber; and (b) of the NIR spectra of the peach fruits. The density plot of (c) 1,440 nm and 1,439 nm; and of (d) 2,172 nm of the NIR spectra

To improve our understanding of this result, the importance of nutrients and FT‐NIR spectra was analyzed by the RF model. The results (Figure 4a,b) showed that the spectral peaks at 1,439, 1,976, and 2,172 nm were significant in the prediction models. The band at 1,976 nm was characteristic of primary amines, and there were various medians within the two clusters (2,172 nm) (Pu Yuan‐Yuan et al., 2020). Furthermore, density plot analysis (Figure 4c) showed that the two clusters were separated at 1,439 and 1,440 nm with different medians. Moreover, the regions identified by the PLS model highlighted the association with the CH group in carbohydrates, consistent with our results for reducing sugar (Kays et al., 2005). Additionally, as shown in Figure 4d, the two nutritive clusters at 2,172 nm showed low overlap with different medians. Thus, these findings suggested that the protein absorption band at around 2,172 nm could be attributed to the combination of C‐O stretching, N‐H bending, and C‐N stretching (Manley & Marena, 2014). Based on these findings, we concluded that the important spectrum at 2,172 nm in our study represented protein. Because protein and reducing sugars were the most important factors in our models, and because we identified the associated spectra, we inferred that our prediction models were accurate and could assess the nutritive qualities of peach fruits using FT‐NIR.

## 4 CONCLUSIONS

In this study, the nutritive qualities of 87 peach fruits were assessed. We classified five nutrients into two clusters; cluster 1 showed low fat and reducing sugar levels, and high protein, crude fiber, and ash levels, whereas cluster two showed high fat and reducing sugar levels, and low protein, cruder fiber, and ash levels, consistent with the results of NIR spectroscopy. We concluded that FT‐NIR spectroscopy of peach fruits could predict the two clusters for the five nutritive qualities. Overall, our findings may help to establish guidelines for promoting the acceptability of peach fruits among consumers.

## CONFLICT OF INTEREST

The authors declare that they have no competing interests.

## Data Availability

The data that support the findings of this study are available from the corresponding author upon reasonable request.
